# ABCDE and ABCDEF care bundles: A systematic review protocol of the implementation process in intensive care units

**DOI:** 10.1097/MD.0000000000014792

**Published:** 2019-03-15

**Authors:** Fabio da Silva Moraes, Lívia Luize Marengo, Marcus Tolentino Silva, Cristiane de Cássia Bergamaschi, Luciane Cruz Lopes, Mariana Del Grossi Moura, Fernando de Sá Del Fiol, Silvio Barberato-Filho

**Affiliations:** a Graduate Program in Pharmaceutical Sciences, University of Sorocaba, Sorocaba, São Paulo; b Faculty of Medicine, Federal University of Amazonas, Manaus, Amazonas, Brazil.

**Keywords:** care bundles, effectiveness, implementation, intensive care units

## Abstract

**Background:**

The awakening and breathing coordination of daily sedation and ventilator removal trials, delirium monitoring and management, and early mobility and exercise (ABCDE) and assessment, prevent and manage pain, both spontaneous awakening and spontaneous breathing trials, choice of analgesia and sedation, assess, prevent and manage delirium, early mobility and exercise, family engagement (ABCDEF) bundles are part of the science of the liberation of the intensive care unit (ICU). There are not enough studies that have evaluated the effectiveness and safety of the implementation of these bundles. This study will analyze the implementation process, estimate their effectiveness and safety, and identify barriers, facilitators and attitudes that have influenced the implementation process.

**Methods:**

Qualitative and quantitative studies will be eligible for our systematic review with adult patients who have been exposed to the implementation of the ABCDE or ABCDEF bundles compared to the usual care in the ICU. In order to search the implementation interventions of the bundles, we will search electronically: MEDLINE (PubMed); Excerpta Medica Database (Ovid); Cumulative Index to Nursing and Allied Health Literature (EBSCO); The Cochrane Library (Wiley); Web of Science; Virtual Health Library; and OpenGrey. We will not impose any language restrictions or publication status. Outcomes of interest include ICU and hospital length of stay; mechanical ventilation time; incidence and prevalence of delirium or coma; level of agitation and sedation; early mobilization; mortality in ICU and hospital; change in perception, attitude or behavior of the stakeholders; and change in knowledge of health professionals. The team of reviewers will independently screen search results, extract data from eligible studies, and assess risk of bias. Disagreements between the reviewers will be solved through consensus or arbitration by a third-party investigator. To assess the quality and risk of bias in randomized and quasi-randomized trials we will use the domain-based evaluation recommended by The Cochrane Handbook. Studies with other methodological designs will be evaluated using the Critical Appraisal Tools developed by The Joanna Briggs Institute. Other instruments may be used, if necessary.

**Results:**

The evidence derived from this study will increase the knowledge of effectiveness and safety of the implementation process of ABCDE and ABCDEF bundles.

**Conclusion:**

The results could guide patients and healthcare practitioners by helping to facilitate evidence-based shared care decision making.

**Protocol registration:**

PROSPERO CRD42019121307.

## 1 Introduction

Over time, a number of changes have occurred in the culture of intensive care and in the management of critical patients, who may be on the front line or at the rear of the process care.^[1]^ During the 1980s and 1990s, the focus was almost exclusively on improving the clinical frontline, for example in the diagnosis and treatment of different forms of shock or in the management of mechanical ventilation.^[2]^

Currently, in addition to addressing the patient's liberation from the intensive care unit (ICU), many researches have extended to the management of the critical care rearguard, in other words, the period after the patient's stabilization, until the last days of hospitalization before his return home.^[3]^

The study of care bundles has also expanded because it is an important tool for obtaining short-, medium- and long-term results, as well as improving care indicators.^[4]^ A care bundle can be defined as “a small set of evidence-based interventions for a defined patient group or population and a care setting that, when implemented together, will bring better results than when implemented individually.”^[5]^

The awakening and breathing coordination of daily sedation and ventilator removal trials, delirium monitoring and management, and early mobility and exercise (ABCDE) bundle represents awakening and breathing coordination exercises, controlling daily sedation and removing mechanical ventilation; the choice of analgesics and sedatives; the monitoring and control of delirium; and mobilization and early exercise.^[6]^

The assessment, prevent and manage pain, both spontaneous awakening and spontaneous breathing trials, choice of analgesia and sedation, assess, prevent and manage delirium, early mobility and exercise, family engagement (ABCDEF) bundle include protocols of spontaneous awakening and spontaneous breathing; choose analgesics and sedatives; evaluate, prevent and manage delirium; promote early mobility and exercise; and involve the family.^[7]^

Protocols and guidelines are important tools for the development of health policies, since they guide planning, delivery, evaluation, and allow the improvement of the quality of health services.^[8]^ Implementation methods can vary widely, depending on the intervention complexity and the theoretical model of knowledge transfer to practice, such as the consolidated framework for implementation research and the engage, educate, execute and evaluate framework. Thus, the implementation process ranges from simple interventions such as dissemination of educational material to the most complex and multifaceted ones like tutorials and consulting sessions.^[9]^

In general, in the field of ICU practices, there is some resistance to change until the proposed concept is consistent and reproducible. It is very common for practitioners to change their practice when the results are consistent across types of studies and populations treated.^[10]^ Thus, the intensivist community needs to be liberated from the key elements of critical patient care in the past, still belonging to today's practices even after 50 years of evidence supporting the need to change.^[11]^

In this context, the present study aims to analyze the implementation process of ABCDE or ABCDEF care bundles in ICU, estimate their effectiveness and safety, and identify barriers, facilitators and attitudes that have influenced.

## 2 Methods

### 2.1 Standards

This review protocol was prepared using the preferred reporting items for systematic reviews and meta-analyses protocol (PRISMA-P) guidelines.^[12]^ The systematic review will be performed and reported according to the PRISMA statement.^[13]^

### 2.2 Protocol and registration

We registered our review protocol with the international prospective register of systematic reviews (PROSPERO) CRD42019121307 (https://www.crd.york.ac.uk/PROSPERO/display_record.php?RecordID=121307). Ethical approval is not required because this is a literature-based study.

### 2.3 Eligibility criteria

#### 2.3.1 Inclusion criteria

Adults patients (>18 years old) admitted to the ICU and exposed to the implementation of the ABCDE or ABCDEF care bundles compared to usual care. The type of study included will be qualitative and quantitative studies describing implementation process.

#### 2.3.2 Exclusion criteria

Congress abstracts; protocols, secondary and tertiary studies; other types of care bundles; evaluation of clinical tools; and institutional guidelines.

### 2.4 Measure outcomes

We will include studies that report any of the following outcomes.

#### 2.4.1 Primary outcomes

- Length of stay in the ICU;
- Mechanical ventilation time;
- Incidence and prevalence of delirium or coma;
- Level of agitation and sedation; and
- Early mobilization.

#### 2.4.2 Secondary outcomes

- Mortality in the ICU and hospital;
- Hospital length of stay;
- Change in perception, attitude or behavior of the stakeholders; and
- Change in knowledge of health professionals.

### 2.5 Search methods for primary studies

We will not impose any language restrictions or publication status.

#### 2.5.1 Electronic searches

We will search the following electronic databases: MEDLINE (PubMed); Excerpta Medica Database (Ovid); Cumulative Index to Nursing and Allied Health Literature (EBSCO); The Cochrane Library (Wiley); Web of Science; OpenGrey (http://www.opengrey.eu/).

#### 2.5.2 Searching other resources

Other studies described in full text and grey literature may be included if they contain sufficient and relevant data. When needed, authors will be contacted for additional information. Reference lists of potentially eligible studies and systematic reviews will be analyzed to identify other relevant studies.

### 2.6 Search strategy

The search strategy will be designed with the assistance of a trained librarian. We will use the following keywords

ABCDE OR ABCDEF OR “PAD guideline” OR “ICU liberation” OR “PAD care bundle,” and methodological filters will be applied to limit retrieval to primary studies. The search strategy will be adapted for each database and details will become available upon completion of the review.

### 2.7 Eligibility determination

Reviewers, working in pairs, will independently monitor potentially relevant citations and abstracts applying the selection criteria. We will obtain full texts of any article that is considered eligible. The same reviewers will independently evaluate the eligibility of each full-text article. In case of duplicate publication, we will use the article with the most complete data. Disagreements between the reviewers will be resolved through consensus or arbitration by a third-party investigator.

### 2.8 Data extraction

The same reviewers, working in pairs, will independently extract the data and will record information regarding patients, methods, interventions, outcomes, and missing outcome data using standardized and pretested data extraction forms with instructions. Before starting data abstraction, we will conduct calibration exercises to ensure consistency between reviewers. We will contact study authors to resolve any uncertainties. Disagreements between the reviewers will be resolved through consensus or arbitration by a third-party investigator.

### 2.9 Risk of bias in individual studies

We will critically appraise included papers, in duplicate and independently, for risk of bias. For randomized and quasi-randomized trials, we will use the domain-based evaluation recommended by The Cochrane Handbook.^[12,13]^ These domains include the following: random sequence generation, allocation concealment, blinding, incomplete outcome data, selective reporting, and other biases. After completing the quality assessment of the 6 domains, we will classify the selected studies into the following categories: low risk of bias (low risk in all domains); high risk of bias (high risk in 1 or more domains); and unclear risk of bias (unclear risk in 1 or more domains). Studies with other methodological designs will be evaluated using the critical appraisal tools developed by The Joanna Briggs Institute.^[14]^ If necessary, other instruments may be used. Disagreements between the reviewers will be resolved through consensus or arbitration by a third-party investigator.

### 2.10 Data synthesis

The implementation strategies refer to the formal steps taken by different institutions to implement ABCDE and ABCDEF care bundles. These strategies will be identified in included studies and classified according to the taxonomy developed by the Cochrane effective practice and organization of care group.^[15]^ This taxonomy provides criteria for different interventions using 4 main domains: organization, finance, governance, and implementation strategies. Information related to barriers and facilitators of the implementation process will be identified and presented in table form.

Whenever possible, for meta-analysis we will quantitatively pool the results at the patient level for the included studies and adopt statistical techniques to manage the difference in study quality and design.

Meta-analyses will be conducted using STATA software (version 14.2). Statistical significance was defined as *P* < .05. The heterogeneity between the studies will be tested using the Cochran *Q* test of heterogeneity and Higgins and Thompson *I*^2^.^[16]^ The degree of heterogeneity was defined as an *I*^2^ value: low (25% to 49%), moderate (50% to 74%) and high (>75%). Provided an appropriate number of studies are eligible for inclusion, we will assess for publication bias using an Egger plot or other statistical techniques. Summary tables will be presented and the narrative summary provided if the meta-analysis is not appropriate due to population heterogeneity, intervention, comparator, outcome or method.

If appropriate, subgroup analysis will explore differences between groups, provided the data presented on literature allow the examination of such, type of care bundle (ABCDE or ABCDEF), number or type of bundle elements implemented, outcomes, for example.

### 2.11 Ethics and dissemination

Ethical approval is not needed for a systematic review that does not involve privacy concerns due to collection or presentation of data from individual patients. The systematic review will be published in a peer-reviewed journal and presented at conferences.

## 3 Discussion

Our review will evaluate evidence of the effectiveness and safety of the implementation process of ABCDE and ABCDEF bundles for adult patients admitted to the ICU also provide estimates of the implementation process and associated risks and evaluate the quality of evidence from qualitative and quantitative studies. The results of our systematic review will be of interest to critical care managers and practitioners around the world.

The information compiled on the implementation processes will inform patients and health professionals about their effectiveness and safety helping facilitate decision making for implementation of bundles in intensive care. This study will also identify gaps for future research.

### 3.1 Strengths and limitations of this study

- This systematic review will assess the effectiveness and safety of implementing ABCDE and ABCDEF care bundles to liberate ICU patients. The method of this review includes explicit eligibility criteria, comprehensive and extensive database research, independent and paired evaluation for study selection.
- We will assess the risk of bias in qualitative and quantitative studies that will be included.
- The quality of the primary studies to be included can be a limiting factor if there are uncontrolled studies, with low sample value and small effect size, and therefore they will be at high risk of bias. These limitations may decrease the quality of the evidence from the study findings regarding the effectiveness and safety of ICU care bundle implementation processes.
- Results can guide intensive care managers and practitioners about the effectiveness and safety of bundle implementation interventions helping facilitate decision making in the critical care setting.

## Author contributions

FSM is the principal investigator and led the writing of the manuscript. SB-F and CCB are the project managers and coinvestigators and contributed to the writing and revision of the manuscript. MTS, LCL, LLM, MDG, and FDF are coinvestigators and contributed to the writing and revision of the manuscript. All authors read and approved the final manuscript.

**Conceptualization:** Silvio Barberato-Filho, Fabio da Silva Moraes, Cristiane de Cássia Bergamaschi.

**Data curation:** Silvio Barberato-Filho.

**Formal analysis:** Fabio da Silva Moraes, Lívia Luize Marengo, Cristiane de Cássia Bergamaschi, Luciane Cruz Lopes, Mariana Del Grossi Moura, Fernando de Sá Del Fiol, Silvio Barberato-Filho.

**Funding acquisition:** Fernando de Sá Del Fiol, Silvio Barberato-Filho.

**Investigation:** Fabio da Silva Moraes, Lívia Luize Marengo, Marcus Tolentino Silva, Cristiane de Cássia Bergamaschi, Luciane Cruz Lopes, Mariana Del Grossi Moura, Fernando de Sá Del Fiol, Silvio Barberato-Filho.

**Methodology:** Fabio da Silva Moraes, Lívia Luize Marengo, Marcus Tolentino Silva, Cristiane de Cássia Bergamaschi, Luciane Cruz Lopes, Mariana Del Grossi Moura, Silvio Barberato-Filho, Cristiane de Cássia Bergamaschi, Luciane Cruz Lopes, Marcus Tolentino Silva.

**Project administration:** Fabio da Silva Moraes, Cristiane de Cássia Bergamaschi, Silvio Barberato-Filho.

**Resources:** Silvio Barberato-Filho.

Silvio Barberato-Filho - https://orcid.org/0000-0001-5179-3125

**Supervision:** Fabio da Silva Moraes, Silvio Barberato-Filho.

**Writing – original draft:** Fabio da Silva Moraes, Lívia Luize Marengo, Marcus Tolentino Silva, Cristiane de Cássia Bergamaschi, Luciane Cruz Lopes, Fernando de Sá Del Fiol, Silvio Barberato-Filho, Cristiane de Cássia Bergamaschi, Marcus Tolentino Silva.

**Writing – review and editing:** Fabio da Silva Moraes, Lívia Luize Marengo, Marcus Tolentino Silva, Cristiane de Cássia Bergamaschi, Luciane Cruz Lopes, Mariana Del Grossi Moura, Fernando de Sá Del Fiol, Silvio Barberato-Filho, Lívia Luize Marengo, Mariana Del Grossi Moura, Fernando de Sá Del Fiol, Luciane Cruz Lopes, Marcus Tolentino Silva.

Fabio da Silva Moraes orcid: 0000-0002-1432-196X.
